# Predictive and Reactive Locomotor Adaptability in Healthy Elderly: A Systematic Review and Meta-Analysis

**DOI:** 10.1007/s40279-015-0413-9

**Published:** 2015-10-20

**Authors:** Sebastian Bohm, Lida Mademli, Falk Mersmann, Adamantios Arampatzis

**Affiliations:** Department of Training and Movement Sciences, Humboldt-Universität zu Berlin, Philippstr. 13, Haus 11, 10115 Berlin, Germany; School of Physical Education and Sports Science, Aristotle University of Thessaloniki, Serres, Greece

## Abstract

**Background:**

Locomotor adaptability is based on the implementation of error-feedback information from previous perturbations to predictively adapt to expected perturbations (feedforward) and to facilitate reactive responses in recurring unexpected perturbations (‘savings’). The effect of aging on predictive and reactive adaptability is yet unclear. However, such understanding is fundamental for the design and application of effective interventions targeting fall prevention.

**Methods:**

We systematically searched the Web of Science, MEDLINE, Embase and Science Direct databases as well as the reference lists of the eligible articles. A study was included if it addressed an investigation of the locomotor adaptability in response to repeated mechanical movement perturbations of healthy older adults (≥60 years). The weighted average effect size (WAES) of the general adaptability (adaptive motor responses to repeated perturbations) as well as predictive (after-effects) and reactive adaptation (feedback responses to a recurring unexpected perturbation) was calculated and tested for an overall effect. A subgroup analysis was performed regarding the factor age group [i.e., young (≤35 years) vs. older adults]. Furthermore, the methodological study quality was assessed.

**Results:**

The review process yielded 18 studies [1009 participants, 613 older adults (70 ± 4 years)], which used various kinds of locomotor tasks and perturbations. The WAES for the general locomotor adaptability was 1.21 [95 % confidence interval (CI) 0.68–1.74, *n* = 11] for the older and 1.39 (95 % CI 0.90–1.89, *n* = 10) for the young adults with a significant (*p* < 0.05) overall effect for both age groups and no significant subgroup differences. Similar results were found for the predictive (older: WAES 1.10, 95 % CI 0.37–1.83, *n* = 8; young: WAES 1.54, 95 % CI 0.11–2.97, *n* = 7) and reactive (older: WAES 1.09, 95 % CI 0.22–1.96, *n* = 5; young: WAES 1.35, 95 % CI 0.60–2.09, *n* = 5) adaptation featuring significant (*p* < 0.05) overall effects without subgroup differences. The average score of the methodological quality was 67 ± 8 %.

**Conclusions:**

The present meta-analysis provides elaborate statistical evidence that locomotor adaptability in general and predictive and reactive adaptation in particular remain highly effective in the elderly, showing only minor, not statistically significant age-related deficits. Consequently, interventions which use adaptation and learning paradigms including the application of the mechanisms responsible for an effective predictive and reactive dynamic stability control may progressively improve older adults’ recovery performance and, thus, reduce their risk of falling.

**Electronic supplementary material:**

The online version of this article (doi:10.1007/s40279-015-0413-9) contains supplementary material, which is available to authorized users.

## Key Points

**Table:** 

Older adults are able to adapt effectively to repeated movement perturbations by applying predictive and reactive motor adjustments.
General locomotor adaptability and predictive and reactive adaptation in particular are not significantly affected by aging.
Fall prevention interventions should consider the repeated application of the mechanisms responsible for an effective predictive and reactive dynamic stability control in order to facilitate adaptation and learning and, thus, to progressively improve older adults’ recovery performance.

## Introduction

During daily locomotion, dynamic stability control of the human body allows for safe and directed movements [1, 2]. However, aging increases the risk of falls [3], particularly, during walking [4, 5]. The combination of a high incidence of falls together with a high susceptibility to injury and the increased severity of consequences [6, 7], poses major threats to the individuals [8], but also an economic burden to the society [9]. Falls are a leading cause of mortality and morbidity in older people (≥65 years) worldwide [10], which makes research into fall prevention an issue of major importance. Although in recent years a vast body of research has focused on this field, fall prevention still remains a rather unsolved challenge, as demonstrated by the incidence of fall-related injuries and deaths that continues to escalate along with the average age of current populations [11].

Motor adaptive behavior is governed by reactive as well as predictive control processes [1, 12, 13], which are required for an appropriate application of stability mechanisms (e.g., modifications of the base of support and/or counter rotation of segments around the body’s center of mass [14]) in order to maintain postural stability during challenging conditions (e.g., perturbations) [15–17]. After a sudden perturbation, the neural system provides appropriate motor commands based on sensory input, in order to execute the necessary reactive postural corrections to recover stability and prevent a fall (i.e., initial feedback-based reactive response) [18]. The age-related degeneration of the human neural [19–24] and musculoskeletal system [25–29] causes a decline in the ability of older adults to re-establish postural equilibrium after a sudden perturbation [16, 30–32].

Error feedback information from such a movement perturbation is used to predictively adapt the locomotion to persisting or recurring perturbations in a feedforward manner [16, 17, 33–35]. Predictive adaptability is typically assessed by means of after-effects, which occur after perturbations when baseline conditions (undisturbed movement) are restored and prior to or during the onset of the expected perturbation [33–35]. After-effects can be seen as an indicator for a specific learning mechanism that enables a sensorimotor recalibration to the experienced perturbation or changed mechanical environment [13, 34]. The effective use of predictive adjustments prepares the system for the upcoming postural threat and can reduce the consequences of the disturbance, making the reactive recovery easier and more successful [15, 16, 36–39].

The recurring experience of perturbations further facilitates the purely reactive response during re-exposure to the same unexpected perturbation [17, 37, 40–42]. This type of motor adaptability can be observed in terms of a reemergence of a learned response after a wash-out (i.e., extinction) phase [13, 43, 44] and improves the effectiveness of the reactive recovery component [17]. In the present article, we termed this specific learning mechanism ‘reactive adaptability’, which is also described as ‘savings’, as an example of ‘meta-learning’ in basic motor learning research [13, 45]. The motor response to repeated expected perturbations involves both predictive and reactive processes and will henceforth be referred to as general locomotor adaptability [40, 46].

With aging, structures in the brain that are associated with movement adaptation (i.e., cortico‐cerebellar [47–53] and cortico‐striatal networks [54, 55]) show degenerative changes [56–60], indicating a potential decline of the adaptive function. However, the effect of aging on the specific predictive and reactive adaptability during locomotion remains elusive. Whereas the results of some studies indicated similar predictive adjustments of older compared with younger adults [16, 61, 62], others identified clear age-related deficits [63, 64]. Similarly, studies that investigated reactive adaptability reported inconsistent findings as well [17, 37, 40, 62]. Bierbaum et al. [17] and Pavol et al. [40] showed significant reactive adaptation of older adults during walking and sit-to-stand; however, the adaptive adjustments tended to be smaller compared with those of younger adults. In contrast, the study from Karamanidis et al. [81] did not find significant reactive adaptation of older participants during disturbed treadmill walking at all.

Since most studies featured only a small number of participants (on average *n* = 25 in the aforementioned studies), used different experimental designs (i.e., type of locomotion and perturbation) and used diverse parameters to quantify adaptive changes, a profound basis for general conclusions on the effect of aging on locomotor adaptability is still missing. Thus, it is yet unclear if an age-related impairment of adaption to repeated movement perturbation exposure (i.e., predictive and reactive locomotor adaptability) might also contribute, along with the decline of the aforementioned initial recovery response, to the higher risk of falling in senescence.

Research on locomotor adaptability is highly important, as it adds insight into the development of stability mechanisms during aging, and may contribute significantly to the design of effective exercise interventions aiming towards fall prevention. Therefore, the objective of the present study was to systematically review literature reports on locomotor adaptability following repeated mechanical perturbations during a broad range of locomotion types to assess the effect of age, differentiated for general, predictive and reactive adaptation. As such, this meta-analysis may provide crucial information on the mechanisms underlying the age-related reduced stability performance, expanding our knowledge on how exercise interventions targeting fall prevention should be designed and applied.

## Methods

### Search Strategy

The search was performed by using the electronic bibliographic databases Web of Science, MEDLINE, Embase and Science Direct (inception to January 2015) and by manually screening the reference lists of the eligible articles. Sets of terms relating to adaptability (adaptation, adaptive, adaptational, adaptability, adjustments, modifications, responses), motor control or behavioral effect (feedforward, feedback, proactive, predictive, reactive, aftereffect, after-effect, after-effects), subjects (old, aged, age, aging, ageing, senior, elderly) and locomotion (walking, walk, gait, run, running, sit-to-stand, stand up, transition, stability, split-belt) were combined in the database search (see Electronic Supplementary Material Appendix S1). Each term was mapped to MeSH (Medical Subject Headings) and controlled terms, respectively.

### Study Selection and Inclusion Criteria

**Fig. 1:**
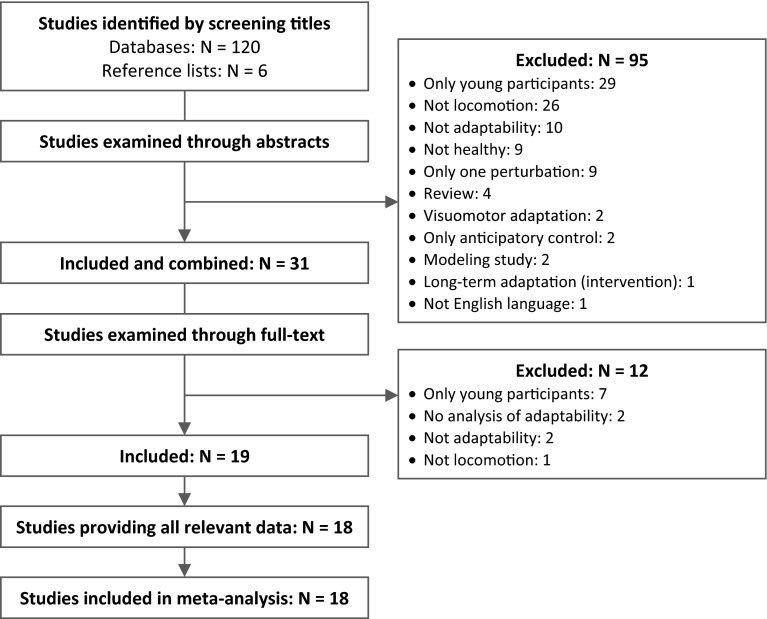
Flowchart of the systematic review process

Two reviewers (S.B. and F.M.) independently evaluated the titles of the studies that resulted from the search, and included studies when the title indicated that the following inclusion criteria were fulfilled: (a) an investigation of the locomotor adaptability and its respective predictive and/or reactive components (e.g., during gait, sit-to-stand, gait termination or initiation, transition, running) of (b) healthy, (c) older adults (i.e., age above 60 years) (d) in response to repeated sudden mechanical movement perturbations (e.g., slips, trips, split-belt). Abstracts and, thereafter, the full-texts were examined to confirm the inclusion. If a study did not fulfill all criteria, the respective exclusion criterion was documented and the study was not considered for further analysis. In the case of disagreement between the two reviewers, a third reviewer (A.A.) was consulted. Figure 1 illustrates the systematic review process of the present meta-analysis. When a study presented the data of different groups, but not all of them fulfilled the inclusion criteria, only the one that met all criteria was included (e.g., healthy control group). Furthermore, intervention studies that provided relevant data in their pre-measurement were considered.

### Data Extraction

Key data from each study were extracted by one reviewer (S.B.) and confirmed by a second one (L.M.). The data were merged in a table, including information on the source (name of the first author and citation), participant characteristics (age, sex and health status), methods (i.e., experimental design, perturbation and protocol), measures and main outcome. If the required outcome data (i.e., means and standard deviations) were not reported in the article or were presented in an inappropriate format for data extraction (e.g., graph instead of values), the corresponding authors were contacted and asked to provide the missing values. Extracting values visually from a graph was the last option. In cases where the relevant data were not available, the study was excluded.

### Statistical Analysis

In order to assess the locomotor adaptability in response to repeated mechanical perturbations, the effect size of the general adaptability was calculated for each study. According to Cohen [65], an effect size of ≤0.49 indicates small, 0.50–0.79 medium and ≥0.80 large effects. To gain a more causal insight to the adaptive process, the predictive and reactive adaptation as components of the general locomotor adaptability were also considered if available in the respective studies. The effect size was calculated as the standardized mean difference (SMD; i.e., difference between pre- and post-adaptation trials divided by the pooled standard deviation of both trials), including an adjustment (Hedges’ adjusted *g*) for small sample bias [66, 67]. For each study, the effect of the locomotor adaptability was calculated as the SMD between the motor feedback response associated with the first unexpected perturbation and the averaged perturbation feedback response of the subsequent adapted movement trials. Predictive locomotor adjustments (i.e., after-effects) were analyzed as differences in the movement behavior at baseline assessment (no disturbances) and after one or more perturbed movements when baseline conditions were again restored (in a subsequent trial or during the ongoing movement, for example, during split-belt walking). Reactive adaptations were calculated as differences of the motor feedback response between the first unexpected mechanical perturbation and a recurring, again unexpected, perturbation. In the case of more than one predictive and/or reactive movement trial that allowed for an analysis, the average value and pooled standard deviation was used. When a study was not focused specifically on locomotor adaptability but provided relevant data, it was also considered in the analysis. Indeed, most of the included studies did not explicitly investigate predictive and/or reactive adaptability, but their applied study design provided the respective information and, thus, allowed us to extract the data and to investigate predictive and reactive adaptive responses. The specific data used for the calculations of each SMD from the single studies are reported as notes in the respective figures. Furthermore, SMDs of the single studies were calculated for those parameters that most adequately reflected the adaptive adjustments, i.e., giving higher priority to global stability parameters rather than local parameters (e.g., stability vs. single joint angle). Depending on the respective scale of the applied measures of stability, SMDs could be negative or positive, but both could indicate a similar adaptive effect (e.g., stability improvement), and thus, negative SMDs were inverted if necessary (reported in the respective figure footnote).

The SMDs from all relevant studies were pooled in a meta-analysis to estimate the weighted average effect size [66–68] of (a) the general locomotor adaptability, (b) the predictive adaptation and (c) the reactive adaptation. We used a random-effects model of the generic inverse variance method, which gives more weight to larger studies (i.e., smaller standard errors) and accounts for heterogeneity of the included studies to calculate the weighted average effect size [66, 69]. To analyze the presence of an overall effect of locomotor adaptability as well as predictive and reactive adaption, a test statistic (i.e., *z* test; null hypothesis: no overall effect of the experience of repeated movement perturbations) was applied [66, 70]. A subgroup analysis that included a difference test [66, 70] was conducted for the factor age group (i.e., older vs. young adults), using the respective control group of young adults if available in the study. To avoid a risk of bias in terms of a repeated inclusion of the same participants, not all studies are present in the meta-analysis. The data reported by Pavol et al. [46], Pai et al. [37], Pavol et al. [40] and Pai et al. [71] were merged and included only once as they refer to the same participants, indicated by the equal number and anthropometrics (i.e., data taken from Pavol et al. [40] and Pai et al. [71]). Furthermore, regarding the studies of Yang and Pai [61], Bhatt et al. [72], Pai et al. [73, 74], only the data from Pai et al. [73] (predictive adaptation) and the data from Pai et al. [71] (locomotor adaptability and reactive adaptation) were considered in the meta-analysis since the experiments of the other studies were based on the same participant pool (as confirmed by one author). The means and standard deviations reported by Pavol et al. [40], Pai et al. [71, 73] were visually extracted from the respective graph. Forest plots were created separately for the general locomotor adaptability as well as predictive and reactive adaptation including the age subgroups, to illustrate the SMDs and 95 % confidence intervals (CIs) for all respective studies as well as the overall effects. Further, heterogeneity between study outcomes was investigated using *Q* and *I*^2^ statistics to assess if differences between outcomes are due to study diversity rather than chance [75]. Statistical procedures as well as forest plots were performed by means of the software Review Manager v.5.3 [76].

### Methodological Qualities and Risk of Bias

A customized methodological quality scale was designed to assess the internal, statistical and external validity of the included studies in regard to the conceptual definition of the present article. The respective items are described in detail in Table 1. A positive point was assigned when the specific quality criterion was fulfilled (Table 1). However, if a criterion could not be scored because it was not part of the study (e.g., no data of predictive adjustments), the criterion was excluded from further quality assessment. The quality score of each validity aspect (i.e., internal, statistical and external) was expressed as number of items with a positive score as a percentage of the total number of items. Thus, 100 % indicates highest possible quality. The single section scores were then averaged to calculate the overall methodological quality of each study. However, a low result in the rating was not an exclusion criterion, but allows for an adequate interpretation of the single study outcomes in the context of the scope of the current article.

**Table 1:** Criteria of the methodological quality

Internal validity	Scoring
1. Study design	A positive point was assigned if the following aspects were considered: 1. Reactive adaptability (i.e., isolated feedback adjustments in response to repeated unexpected perturbations) 2. Predictive adaptability (i.e., feedforward adjustments based on prior experience) 3. Young control group
2. Methods	A positive point was assigned if the following aspects were considered: A. A number of trials ≥5 for sufficient adaptive improvements [17, 40] B. A standardized perturbation was used to stimulate adaptation [e.g., same leg, same movement characteristics (e.g., velocity), constant perturbation] C. A sufficient perturbation was used to evoke adaptation D. The effect of the security system (e.g., recovery with harness assistance) was controlled
2.1 Reactive	A. A wash-out (i.e., extinction training) phase to avoid the effect of prediction [17] B. The effect of prediction was controlled [17]
2.2 Predictive	A. A perturbation was expectable B. Assessment of after-effects (i.e., return to baseline conditions) [33, 35] C. Assessment prior to/at onset of the potential perturbation [16, 109]
3. Cofactors	A positive point was assigned if the following aspects regarding the participants were considered: A. Influence of sex B. Influence of physical activity level C. Influence of health status D. Influence of cognitive ability [110, 111]

Statistical validity	Scoring
4. Statistical tests	A positive point was assigned if appropriate statistical tests were used
5. Power analysis	A positive point was assigned if effect sizes were calculated and reported

External validity	Scoring
6. Eligibility of sample and variable	A positive point was assigned if the intervention included: 1. An appropriate participant sample (i.e., sample size* n* ≥ 10 and sufficiently representative of the basic population in terms of anthropometrics, health and cognitive status, and activity level) 2. Appropriate variables (adequate indicator for a relevant aspect of motor control, e.g., stability state)
7. Description of the experimental protocol	A positive point was assigned if the following criteria were reported: A. Type of movement B. Movement characteristic (e.g., walking velocity) C. Description of the perturbation (e.g., slip distance) D. Participant instruction E. Number of trials and blocks
8. Description of the participant sample	A positive point was assigned if the following criteria were reported: A. Age B. Sex C. Body height D. Body weight E. Activity level F. Health status G. Cognitive status

The risk of bias in individual studies (sequence generation, allocation concealment, blinding outcome assessor, incomplete outcome data, selective outcome reporting, other sources of bias) was assessed according to the Cochrane Risk of Bias tool [77].

The data extraction and scoring was performed by two independent observers (L.M. and S.B.), and in the case of disagreement, a third one was consulted (A.A.).

## Results

### Literature Search

Figure 1 illustrates the systematic search process. The search strategy yielded 2023 hits in the four databases. After screening study titles and eliminating duplicates, 120 potentially eligible studies were identified. Following the abstract examination, 25 studies remained included. Twelve of these did not confirm all criteria following the review of the full texts and, thus, were excluded from the further analysis. The reference list search of the included studies provided six further related studies. One study was excluded from the 19 studies identified because of unavailable data [78]. Finally, 18 studies fulfilled all criteria and were included in the present meta-analysis (Fig. 1).

### Description of the Included Studies

The present systematic review included in total 18 studies (participants in total *n* = 1009) eligible for the research question, and their characteristics are summarized in Table 2. Seventeen of the 18 studies allowed for the investigation of the locomotor adaptability of older adults in response to repeated mechanical perturbations of different kinds of locomotion. Twelve studies could be used to assess the predictive adaptation component of locomotor adaptability and five for the reactive adaptation component. Locomotor adaptability was mainly investigated during walking (trail, *n* = 9; treadmill, *n* = 5), but also during sit-to-stand (*n* = 4) and gait initiation (*n* = 1). Mechanical movement perturbations were induced by means of slips (*n* = 9), trips (*n* = 4), split-belt walking (*n* = 3), obstacles (*n* = 1) or step target shifts (*n* = 1). Various protocols and parameters were used to quantify adaptive adjustments (listed in Table 2). From the 18 included studies, 12 provided a control group of young adults. The mean age of the 613 healthy older adults of all studies was 70 ± 4 years, and for the 396 young adults, it was 25 ± 1 year. In the 15 studies that reported the sex distribution of their older participants, in total 267 were female and 243 male. The number of young and older participants within studies ranged from 73 to nine, with a mean of *n* = 31 ± 22.

**Table 2:** Summary of included studies. Information were extracted with regard to the effect of locomotor adaptability (i.e., difference between the recovery response following an unexpected perturbation and the response after subsequent movement perturbations) and its distinct predictive (i.e., difference prior to/at the potential perturbation between baseline and an after-effect condition) and reactive (i.e., difference between the recovery response following an unexpected perturbation and the response after an again unexpected perturbation) components

Study	Participants^a, b^	Method	Measure	Outcome
Bhatt et al. [72]	O: Single session: *n* = 25 (73 ± 5 years), 13 F Dual session: *n* = 13 (70 ± 5 years) 7 F Healthy ([61, 72–74] same pool)	Design: Disturbed trail walking (same as in [61, 72–74]) Perturbation: Slip [i.e., hidden free moveable platforms (90 cm forward, 58 cm backward)] Protocol: Baseline, 8 slips, 3 non-slips, 8 slips, 3 non-slips, 15 mixed trials	Parameters: Stability, fall incidence, loss of balance, limb support Magnitude of stability at TD slipping foot (predictive) and magnitude of stability and limb support at lift-off trailing limb (LA) as well as fall incidence and loss of balance between the baseline or first slip trial and last slip trial	The older adults significantly reduced their fall incidence and loss of balance (LA) and increased their pre-slip (predictive) and post-slip stability (LA) by the end of the session
Bierbaum et al. [16]	O: *n* = 13 (67 ± 3 years), 0 F Y: *n* = 10 (26 ± 3 years), 0 F Active, healthy	Design: Disturbed trail walking (60 % of walk-to-run transition velocity) Perturbation: Trip (i.e., change hard to soft surface) Protocol: Baseline followed by adaptation phase with 19 trials on soft or hard surface (2nd, 8th and 19th)	Parameters: Margin of stability (and components), base of support, GRF Rate and magnitude of LA (TD recovery leg) and predictive adjustments (TD disturbed leg)	Despite an age-related reduced recovery performance after the first unexpected perturbation, young and older adults showed similar significant LA and predictive stability control. Predictive adjustments were present directly after the first perturbation trial in both age groups. Adaptive motor adjustments (LA) improved over consecutive trials
Bierbaum et al. [17]	O: *n* = 14 (67 ± 4 years), 0 F Y: *n* = 14 (25 ± 2 years), 0 F Active, healthy	Design: Disturbed trail walking (60 % of walk-to-run transition velocity) Perturbation: Trip (i.e., change hard to soft surface) Protocol: Baseline followed by 5 unexpected perturbation trials, each after 4–8 unperturbed trials (wash-out phase)	Parameters: Margin of stability (and components), base of support Rate and magnitude of reactive adjustments	Reactive adaptive adjustments significantly improved over the 5 unexpected perturbations. Young adults showed a tendency towards greater reactive adaptability compared with older adults
Bohm et al. [36]	Control group O: *n* = 14 (70 ± 4 years), 0 F Y: *n* = 15 (26 ± 3 years), 0 F Active, healthy	Design: Disturbed trail walking (self-selected speed) Perturbation: Trip (i.e., change hard to soft surface) Protocol: Baseline followed by adaptation phase with 15 trials on soft or hard surface (2nd, 7th and 13th) (comparable to [16])	Parameters: Margin of stability (and components), base of support Rate and magnitude of LA (TD recovery leg) and predictive adjustments (TD disturbed leg)	Young and older adults showed similar significant LA and predictive stability control. Predictive adjustments were present directly after the first perturbation trial in both age groups. Adaptive motor adjustments (LA) improved over consecutive trials
Bruijn et al. [63]	O: *n* = 12 (73 ± 5 years), 3 F Y: *n* = 8 (22 ± 4 years), 4 F Healthy	Design: Disturbed treadmill walking Perturbation: Different speeds of the belts (0.5 and 1.0 m/s) Protocol: Tied-belt (baseline, 5 min), split-belt (10 min), tied-belt (after-effect condition, 5 min)	Parameters: Step length symmetry, stride length, swing time, swing speed Rate and magnitude of adaptation to split-belt condition (LA) and after-effect condition (predictive)	The older adults adapted less and more slowly to split-belt walking (LA) and showed fewer after-effects (predictive adaptation) than young adults
Chambers and Cham [64]	O: *n* = 9 (60 ± 4 years), ? F Y: *n* = 11 (23 ± 2 years), ? F Healthy	Design: Disturbed trail walking (self-selected speed) Perturbation: Slip (i.e., slippery solution on the surface) Protocol: Baseline followed by unexpected slip and 5 consecutive non-slip trials (alert dry)	Parameters: Activation (EMG, onset, duration, power, co-contraction index) of lower limb muscles during stance phase (heel contact to toe off) Magnitude and time of muscle activation at heel contact and toe off during baseline and alert dry trials (predictive)	The muscle activation following the unexpected slip was scaled to slip severity. The younger adults showed a more powerful and longer activity. Young and older adults similarly presented a more powerful muscle activation and co-contraction at the ankle and knee as well as earlier onsets and longer durations in the posterior muscles during the alert dry trials. These predictive changes were partly enhanced in the young adults
Van Hedel and Dietz [112]	O: *n* = 9 (63 ± 7 years), 2 F, (−1) Y: *n* = 9 (23 ± 3 years), 2 F Healthy	Design: Disturbed treadmill walking Perturbation: Obstacle (i.e., foam stick at speed of the walking velocity, task: stepping over obstacle as low as possible) Protocol: Blocks of trails (3 × 50 steps, ~12 min, 5-min breaks) with acoustic signal of upcoming obstacle (every 6–11 steps) and acoustic feedback of foot clearance after passing the obstacle	Parameters: Obstacle hits, foot-obstacle clearance, muscle activity (EMG) of lower limbs, joint angles, swing phase duration Step adjustments (LA) over the obstacles [first 4 steps (onset) vs. last 4 steps (end)] in the same block and between trial blocks (block 1 vs. 2 vs. 3)	Young and older adults presented similar significant LA of stepping performance (foot-obstacle clearance) within the first block while the younger had less obstacle hits. Muscle activity decreased in both age groups, however, significantly only in the elderly. Joint angles and swing phase remained unaffected
Karamanidis et al. [81]	O: *n* = 11 (62–76 years), 11 F Y: *n* = 11 (22–30 years), 11 F	Design: Disturbed treadmill walking (self-selected speed) Perturbation: Trip (i.e., external resistance on the right leg during swing phase) Protocol: Baseline, unexpected trip and 6 recovery steps followed by unperturbed wash-out phase, 11 consecutive disturbed steps and a final undisturbed step (after-effect condition)	Parameter: Margin of stability, base of support Rate and magnitude of LA and predictive adjustments at TD	Although older adults needed more steps to recover after the unexpected trip compared with the young adults, they preserved their LA following consecutive step perturbations. However, responses were delayed compared with young adults. After-effects were unaffected by age (predictive adaptation)
Pai et al. [37]	O: *n* = 41 (73 ± 5 years), 21 F Healthy (same as [37, 40, 46, 71])	Design: Disturbed sit-to-stand (‘stand up as quick as possible’) Perturbation: Slip (i.e., 24 cm forward slide of the surface) Protocol: Baseline trials followed by 5 slips and a reslip after 3–4 non-slip trials (same as in [37, 40, 46, 71])	Parameter: Stability, fall incidence, loss of balance Magnitude of stability at seat-off (predictive) as well as fall incidence and loss of balance (LA)	Older adults significantly reduced their risk of falling and balance loss (LA) attributable to improved predictive motor adjustments
Pai et al. [71]	Walking: O: *n* = 38 (71 ± 5 years), 19 F; Y: *n* = 35 (26 ± 5 years), 18 F ([61, 72–74] same pool) Sit-to-stand: O: *n* = 41 (73 ± 5 years), 21 F; Y: *n* = 60 (25 ± 5 years), 44 F (same as [37, 40, 46, 71])	Walking: Design, perturbation and protocol see Bhatt et al. [72] (same as in [61, 72–74]) Sit-to-stand: Design, perturbation and protocol see Pai et al. [37] (same as in [37, 40, 46, 71])	Parameter: Stability, fall incidence, loss of balance and limb support Magnitude of LA of stability and limb support (300 ms after slip onset) and associated fall incidence and loss of balance	Older adults fall over twice as likely as young adults following the first unexpected slip in both tasks. Both age groups rapidly adapted in walking and sit-to-stand task by improved control of stability and limb support (LA), leading to a significant reduction of falls and balance loss after 5 slips
Pai et al. [73]	O: *n* = 73 (≥65 years), ? F Healthy ([61, 72–74] same pool)	Design, perturbation and protocol see Bhatt et al. [72] (same as in [61, 72–74])	Parameter: Stability, fall incidence Magnitude of stability at TD (30–50 ms prior slip) slipping foot (predictive) and at TD (300–500 ms after slip onset) trailing foot (LA and reactive) as well as fall incidence between the first and last slip trial	Significantly reduced fall rate based on significant LA due to predictive as well as and reactive stability adjustments after the slip and non-slip trials
Pai et al. [74]	O: *n* = 67 (72 ± 6 years), 44 F Healthy ([61, 72–74] same pool)	Design, perturbation and protocol see Bhatt et al. [72] (same as in [61, 72–74])	Parameter: Fall incidence Fall incidence of the first slip trial and last slip trial (LA)	Reduced fall incidence comparing the first with the last slip trial (indicating LA)
Pavol et al. [46]	O: *n* = 41 (73 ± 5 years), 21 F Y: *n* = 60 (25 ± 5 years), 44 F, (−7) Healthy (same as [37, 40, 46, 71])	Design, perturbation and protocol see Pai et al. [37] (same as in [37, 40, 46, 71])	Parameter: Fall incidence, recovery step (occurrence, direction, number) Fall incidence and recovery step characteristics of the first compared with the last trial of young and older adults	Although older adults fell more frequently following the first unexpected perturbation, the fall incidence decreased with repeated slip exposure similarly in both age groups, which was accompanied by changes of the recovery step (LA)
Pavol et al. [40]	O: *n* = 41 (73 ± 5 years), 21 F, healthy Y: *n* = 60 (25 ± 5 years), 44 F, (−9) (same as [37, 40, 46, 71])	Design, perturbation and protocol see Pai et al. [37] (same as in [37, 40, 46, 71])	Parameter: Position and velocity of CoM at seat-off (predictive) Falls, recovery step (length, duration and direction), extrapolated CoM position and hip height (step TD) (LA and reactive) Rate and magnitude of LA (slip 1–5) and magnitude of predictive and reactive (slip–reslip) adjustments	Young and older adults adjusted their CoM position and velocity during seat-off after the 5 perturbation trials (i.e., similar predictive motor adaptations) and therewith contributed to a decrease of fall incidence and changes of recovery step incidence and direction. Predictive and reactive adjustment magnitudes were greater in the young adults
Roemmich et al. [62]	O: *n* = 15 (65 ± 8 years), ? F Y: *n* = 15 (23 ± 2 years), ? F Healthy	Design: Disturbed treadmill walking Perturbation: Different speeds of the belts (100 % and 50 % of fastest comfortable speed) Protocol: Tied-belt (baseline), split-belt for 10 min (early: mean of first 5 steps; mid: mean of 5 steps after 5 min; late: mean of last 5 steps), wash-out, split-belt for 2 min (readapt: mean of first 5 steps) and again tied-belt (post-tied: mean of 5 steps)	Parameter: Step length, stride length and stance time asymmetry Intra-limb (stride length and stance time asymmetry) and inter-limb (step length asymmetry) LA as well as predictive (baseline vs. post-tied) and reactive (early vs. readapt) adaptive changes	Similar predictive and reactive adaptive responses of young and older adults to the sequence of tied- and split-belt walking
Sakai et al. [113]	O: *n* = 45 (71 ± 4 years), 26 F Healthy	Design: Disturbed treadmill walking (2 km/h) Perturbation: Slip (i.e., decelerating right belt for 500 ms at TD of the heel) Protocol: 20 perturbations repeatedly in a 5-min walk	Parameter: Sway, muscle activity (EMG) of lower limbs and trunk, stride time Magnitude of LA as difference between average of 10 early (first half) and 10 late (second half) subsequent perturbations steps	Older adults showed reduced sway (i.e., more stable) in the second half of 20 disturbed steps (LA). While muscle EMG latencies were unchanged, two muscles of the limb indicated reduced EMG magnitude in the second half
Tseng et al. [114]	O: *n* = 18 (72 ± 4 years), 12 F Y: *n* = 36 (26 ± 4 years), 13 F Healthy	Design: Disturbed stepping movements (‘step fast and accurate’) Perturbation: Left or right shift of visual step target during volitional step initiation Protocol: 20 baseline steps followed by a block of 30 adaptation trials (target shift)	Parameter: Step accuracy (foot position), duration (total, response time, weight transfer, stepping execution) Magnitude of LA as difference of early (first 3 of 30 steps) and late adaptation trials (last 3 of 30 steps)	Older adults adapted stepping accuracy almost equivalent to young adults but showed slowness during the stepping movement in the early adaptation phase. With practice, older adults reduced their movement times to levels similar to young adults
Yang and Pai [61]	O: *n* = 73 (73 ± 5 years), 46 F ([61, 72–74] same pool)	Design, perturbation and protocol see Bhatt et al. [72] (same as in [61, 72–74])	Parameter: Stability, fall incidence, analysis of gait pattern, kinematic (trunk, knee, foot) Magnitude of stability, gait pattern and kinematics at TD slipping foot (predictive) and at lift-off trailing limb as well as fall incidence (LA) between the first and last slip trial	Older adults improved gait stability by forward positioning of their CoM in relation to their base of support [shorter steps and forward trunk leaning and flat foot landing with knee flexed (LA and predictive)] following the trial session (first vs. last trial)

*CoM* center of mass, *EMG* electromyography, *F* female, *GRF* ground reaction force, *LA* locomotor adaptability, *O* older adults, *TD* touchdown of respective foot, *Y* young adults, *?* not reported

^a^Age data are mean ± SD or range

^b^Negative numbers in parentheses indicate numbers of participants who were excluded during the course of the study (e.g., due to technical issues)

### Meta-Analysis of Adaptability Effects

**Fig. 2:**
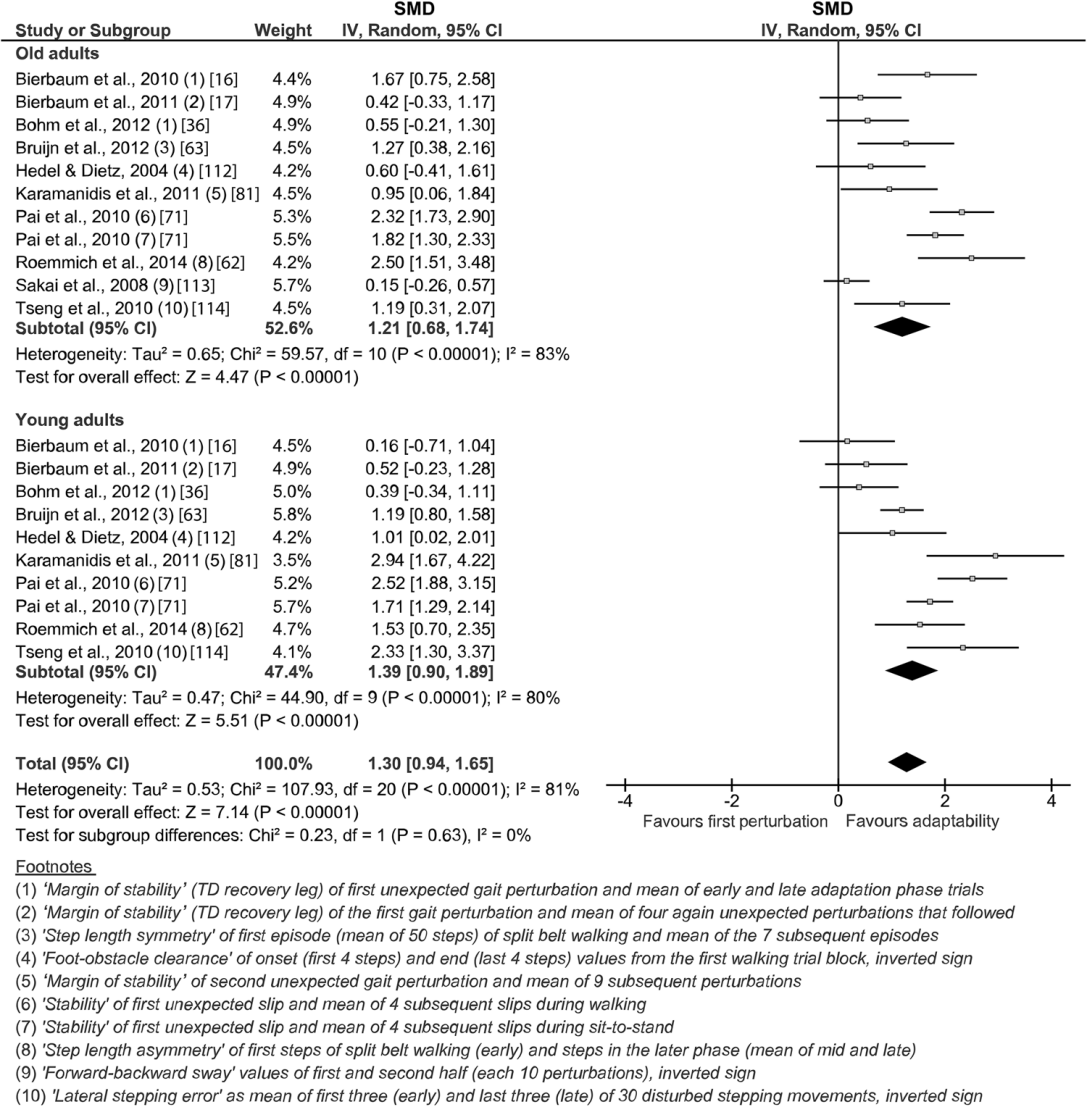
Forest plots for the meta-analysis of human locomotor adaptability in response to repeated perturbations. The general adaptive potential displayed here includes the predictive and reactive components. The *footnotes* explain the data from the original study used for the present analysis. *CI* confidence interval, *IV* inverse variance, *SMD* standardized mean difference, *TD* touchdown

The weighted average effect size of the general locomotor adaptability from the included studies was 1.21 (CI 0.68–1.74, *n* = 11) for the older and 1.39 (CI 0.90–1.89, *n* = 10) for the young adults (Fig. 2). The overall effect was significant (*p* < 0.05) for both age groups, respectively. No statistically significant subgroup differences (i.e., older vs. young) were found in the analysis (Fig. 2), indicating that the experience of repeated mechanical movement perturbations induced similar adaptive recovery responses in both age groups.

**Fig. 3:**
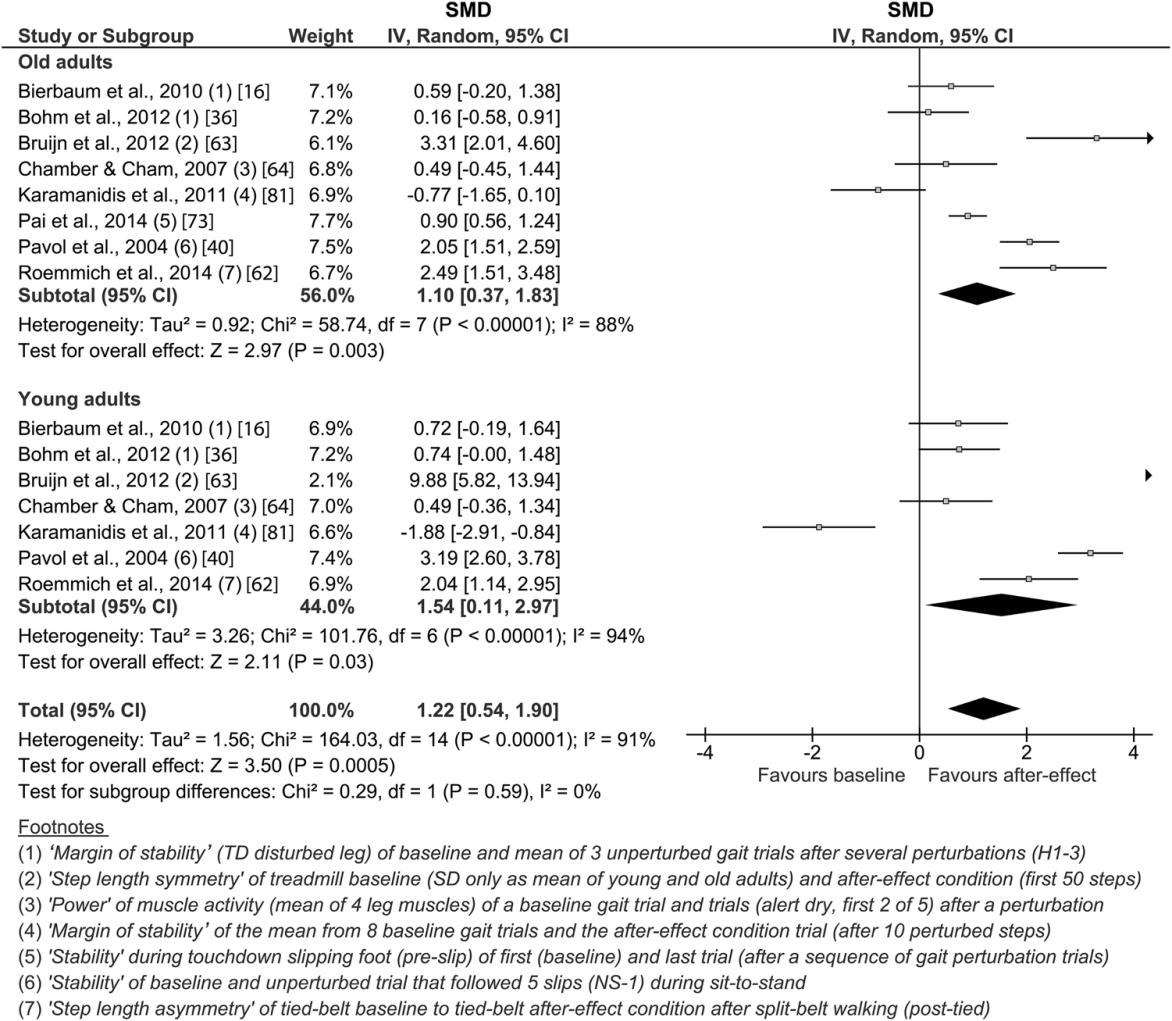
Forest plot of the effect of predictive adaptation on locomotion. The *footnotes* explain the data from the original study used for the present analysis. *CI* confidence interval, *H* hard surface (unperturbed), *IV* inverse variance, *NS-1* non-slip trial (unperturbed), *SD* standard deviation, *SMD* standardized mean difference, *TD* touchdown

For the predictive adaptation, the older adults showed a weighted average effect size of 1.10 (CI 0.37–1.83, *n* = 8) and the young adults one of 1.54 (CI 0.11–2.97, *n* = 7; Fig. 3). The overall effect was significant (*p* < 0.05) for the group of young as well as older adults, but no significant difference was detected between subgroups (i.e., older vs. young).

**Fig. 4:**
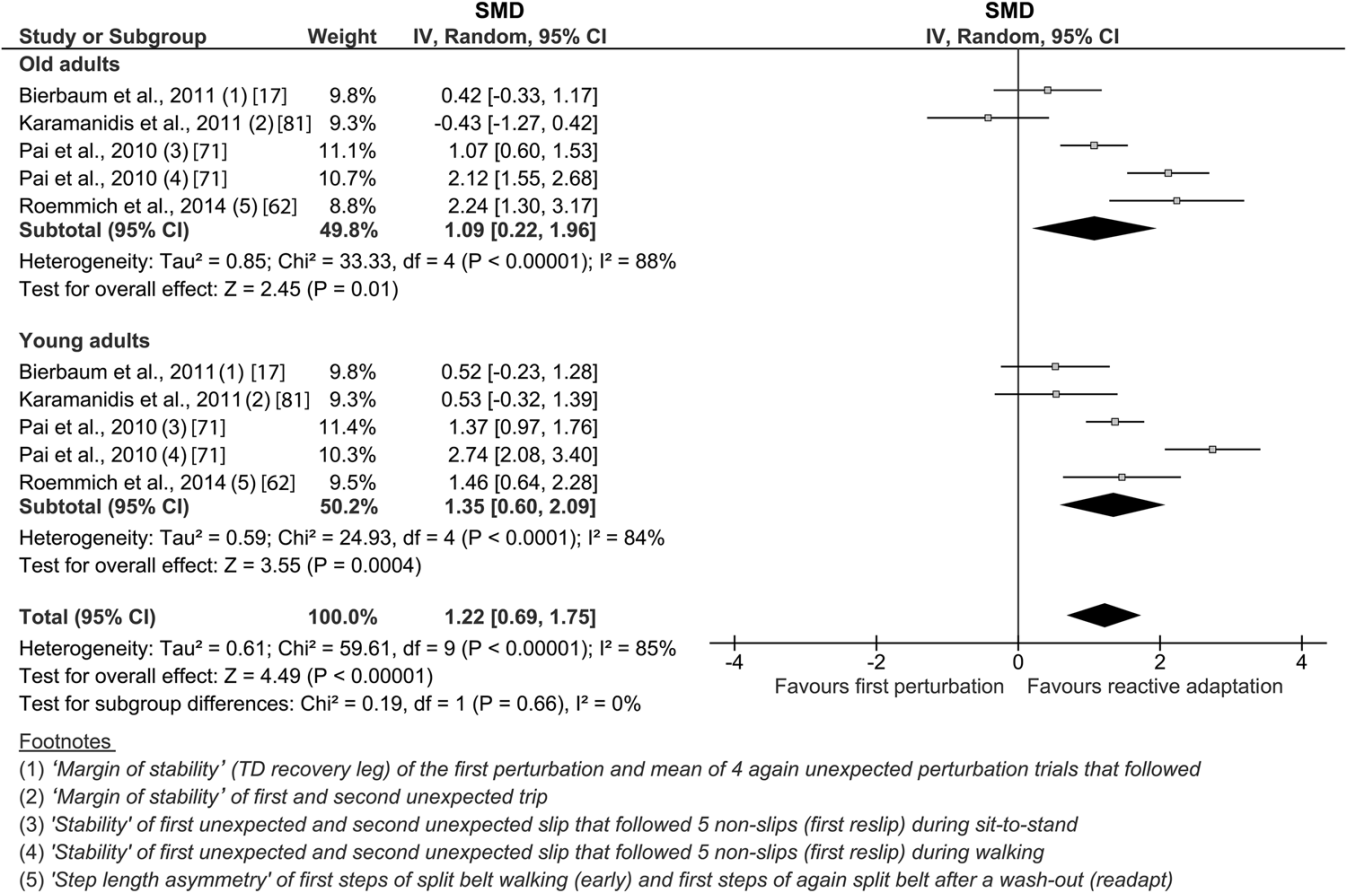
Forest plot of the effect of reactive adaptation on the response to repeated unexpected locomotor perturbations. The *footnotes* explain the data from the original study used for the present analysis. *CI* confidence interval, *IV* inverse variance, *SMD* standardized mean difference, *TD* touchdown

The analysis of the reactive adaptation revealed a weighted average effect size of 1.09 (CI 0.22–1.96, *n* = 5) for the older adults and 1.35 (CI 0.60–2.09, *n* = 5) for the young adults (Fig. 4). The overall effect was significant (*p* < 0.05) for the older and young adults, and no significant group differences were found.

### Methodological Quality and Risk of Bias Assessment

The results of the methodological quality assessment of the included studies are presented in the Electronic Supplementary Material, Table S1, and showed an achieved mean total score of 67 ± 8 % (i.e., internal validity 60 ± 16 %, statistical validity 53 ± 11 %, external validity 89 ± 6 %), indicating appropriate methodological qualities for most studies with regard to the scope of the present meta-analysis. Out of the total number of 17 studies that could be used to assess the general locomotor adaptability, 11 studies further provided data of predictive adaptation and five of these provided data of reactive adaptation. One further study provided information only about predictive and not general or reactive adaptation.

The risk of bias assessment indicated a low risk of bias within studies (Electronic Supplementary Material, Table S1). However, the sequence and allocation domain was not applicable since the present meta-analysis only analyzed a single group at different time points. Furthermore, judgment of the blinding of the assessor to the data was unclear since respective information was not reported in any study.

## Discussion

The present systematic review and meta-analysis assessed locomotor adaptability in response to repeated mechanical movement perturbations in general and predictive and reactive adaptation in particular with respect to the effect of aging. Eighteen studies, with a total number of 1009 participants (613 older adults), were included. The weighted average effect size for the locomotor adaptability was 1.21 for the older and 1.39 for the young adults, with a significant overall test of both groups but no significant age-group differences. The large effect sizes of the examined studies provide evidence that older adults are able to adapt to repeated mechanical locomotor perturbations similar to young adults. Furthermore, the detailed analysis of the predictive and reactive adaptation revealed similarly large weighted average effect sizes, with significant overall effects for both age groups. Although the values were smaller for the older adults, no statistically significant age-group differences were found. These findings suggest that both the predictive as well as reactive adaptation component of locomotor adaptability remains highly effective in older adults.

Adaptation effects were reported consistently in all included studies, applying different types of locomotion paradigms (i.e., trail and treadmill walking, sit-to-stand, gait initiation) and mechanical perturbation types (i.e., slips, trips, split-belt, obstacles, step target shifts) and magnitudes (Fig. 2). The high weighted average effect size of 1.21 for the adaptability of the older adults together with the lack of statistical significant difference compared with the young demonstrates that the ability to adapt to different kinds of repeated mechanical perturbations is not significantly compromised by aging. Locomotor adaptive adjustments improve the recovery performance following subsequent disturbances and may decrease the risk of falls and, thus, fall-related injuries [79]. For example, several of the included studies showed a reduction of the fall incidence of older adults of over 40 % following a novel mechanical perturbation in the last perturbation trials compared with the first exposure after a sequence of slip and non-slip trials [46, 71–74], which was comparable with the young controls [46, 71]. Notably, some studies provided evidence that stability performance can be facilitated [42, 72, 80] for up to 12 months [73] following a short-term (single session) adaptation paradigm, indicating persistent storage of task-relevant information within the motor system (i.e., long-term retention). Furthermore, such adaptive adjustments decreased older adults’ annual risk of fall by 50 % (i.e., effective transfer to daily life condition) [74]. Therefore, our findings of preserved adaptability in the older adults together with the aforementioned reports of associated long-term retention and transfer strongly emphasize the applicability and effectiveness of interventions that incorporate aspects of locomotor adaptation for the age-related prevention of falls.

Locomotor adaptability includes predictive and reactive adaptation [16, 17], which can both account for the increase of the effectiveness of the recovery response to repeated movement mechanical perturbation exposure [40, 46]. Predictive adaptation (demonstrated by after-effects) is present prior to or during the onset of an expected perturbation [33–35] and improves the stability state of young as well as older adults, preparing for the upcoming postural threat and, thus, reducing the consequences of the expected perturbation [15, 16, 36–39]. However, whereas some researchers reported comparable predictive adaptation of young and older adults [16, 61, 62], others identified deficits of the older participants [63, 64]. The current meta-analysis of the included studies revealed large weighted average effect sizes of predictive adaptation for the older (i.e., 1.10) and young (i.e., 1.54) adults with a significant overall test and no statistically significant age-group differences. Therefore, the potential for predictive adaptation seems not to be affected by aging, although the lower weighted average effect size of the older adults may indicate minor age-related deficits. Independent from age, the observed predictive adjustments occurred immediately after the first unexpected mechanical perturbation and were associated with fast improvements of the perturbation recovery response [16, 36, 37, 40]. For normal trail walking, the study from Bierbaum et al. [16] reported that predictive adjustments optimize over several trials in young as well as older adults and that an increase of the base of support was the main mechanism to achieve a more stable body position prior to the expected trip. The latter mechanism was also effective during an experimental treadmill paradigm that applied trips [81]. A prominent predictive adjustment during repeated slipping perturbation in older adults is the shortening of step length at touchdown prior to the expected perturbation and a change of hip, knee and ankle kinematics, most likely to reduce the slip velocity, i.e., its severity [61]. Using a split-belt treadmill paradigm, older adults presented significant after-effects as well [62, 63]. The study of Bruijn et al. [63] reported that the adjustments of the older adults tended to be smaller compared with the young adults, while Roemmich et al. [62] did not find an effect of age. The first of these studies [63] in particular featured a very high SMD for young adults, which may have biased the findings of the current analysis (Fig. 3). However, an exclusion of this study from the analysis resulted in similar results, albeit with lower values (i.e., the weighted average effect size was 0.85 in older adults and 0.91 in young adults, with no difference between groups). The study by Chambers and Cham [64] presented evidence that kinematic and kinetic predictive adjustments are based on adapted activities of the lower limb muscles. In their experiment, the young and older participants presented an increase of the activation and co-contraction of ankle and knee muscles, suggesting a preparation for the expected gait disturbance, though more pronounced in young adults [64]. Regarding adaptations during a sit-to-stand task, predictive adjustments were reported as changes in the center of mass position and velocity in both age groups, which allowed for a greater stability prior to seat-off. As with walking, these changes improved over several slip exposures [37, 40]. In summary, the results of the systematic review and meta-analysis provide evidence for an effective predictive adaptability of older adults with similar motor adjustments compared with younger adults. The specific characteristic of the predictive motor response is related to the locomotor task (e.g., increase or decrease of the base of support), but generally occurs rapidly (i.e., after one perturbation) and improves over time.

Besides predictive adaptation, the reactive response to a mechanical perturbation improves with repeated exposure as well, and is, therefore, a crucial component of locomotor adaptability. Although the underlying mechanism of this specific motor learning aspect is not yet completely understood, the re-exposure to previously experienced perturbations is accompanied by a facilitation of the reactive responses and was, therefore, also referred to as ‘savings’ in basic motor learning research [13, 45]. Due to the small body of relevant literature and inconsistent findings, it was yet unclear if reactive locomotor adaptation is affected in the elderly population [17, 37, 40, 62]. The analysis of the reactive adaptation potential in the current meta-analysis demonstrated large weighted average effect sizes of 1.09 for the older adults and 1.35 for the young adults, with a significant overall effect for both groups, but no statistically significant age-group differences. This means that older adults are able to improve their recovery motor response over several unexpected movement perturbations, showing only minor age-related deficits. However, the results should still be interpreted with care, as the sample size for this part of the analysis was small (i.e., five studies) and the assessment methodology is challenging. For example, it needs to be ensured (e.g., by a wash-out/extinction phase) that no predictive adjustments affect the consequences of the perturbation and, thus, the assessment of the reactive adaptation. The study from Bierbaum et al. [17] is to date the only study that exclusively assessed reactive locomotor adaptation with appropriate methodological quality, and the results reflect the findings of the meta-analysis. The older adults presented significant reactive adaptability in the course of five unexpected gait disturbances; however, there was a tendency towards less prominent adjustments compared with young controls. The authors concluded that this deficiency in reactive adaptability may contribute to the age-related increased risk of falling [17]. Similar results were reported during gait perturbations induced by slipping. Although the older participants presented significant improvements of the reactive adjustments, these were slightly smaller compared with young adults [71]. During split-belt treadmill walking, older adults reduced their step length asymmetry to a greater extent than younger ones, most likely as a consequence of a greater asymmetry during the initial split-belt condition [62]. The study from Karamanidis et al. [81] was the only study that showed no reactive adaptability during disturbed treadmill walking of the older adults in contrast to the young controls. While the margin of stability in the post-adaptation condition increased for the young participants (i.e., indication for reactive adaptation), it decreased for the older adults. Several of the included studies investigating sit-to-stand tasks reported reactive adaptive changes in the recovery stepping behavior as well [37, 40, 46]. Some of the participants adjusted their reactive response by implementing a recovery step in the second unexpected slip (i.e., post condition) and participants who already used a step in the initial recovery response improved the positioning of the step (i.e., more posterior) and, therewith, enhanced the body’s stability state [40]. Persistent age-group differences across trials indicated that this reactive adaptive behavior was less effective for the older adults compared with younger ones [40]. Taken together, our meta-analysis demonstrated the presence of reactive adaptability in older adults. As discussed, some of the included studies reported potential age-related deficits that may account for the lower effectiveness of the adjustments and accordingly explain the lower weighted average effect size of the older adults. The controversial results of greater adaptive responses of the older compared with young adults [62] or, to the contrary, absence of reactive adaptive responses [81] may indicate a certain relation of the extent and characteristic of the specific adaptive adjustment to the locomotor task and the respective mechanical perturbation type and magnitude.

The most important outcome of the present analysis is that locomotor adaptability and especially predictive and reactive adaptation persist in the elderly population. Following an unexpected mechanical movement perturbation (i.e., purely reactive and without preceding adaptation), older adults demonstrate large deficits in their recovery performance compared with young adults [16, 36, 46]. Various physiological age-related impairments may cause this less effective initial feedback response, e.g., decline of muscle-tendon capacities [29, 82–84], inappropriate application of mechanisms responsible for dynamic stability control [85], reduced range of motion [86], impairments of the central [19, 20] and peripheral [21–24, 87] nervous system capacity, and limited cognitive and attentional capabilities [88, 89]. The present findings of preserved adaptability in older adults provide indirect evidence that while age-related increased risk of falling is associated with a reduced ability to perform online motor corrections necessary to maintain stability after a sudden unexpected perturbation (thereby avoiding a fall), the adaptive potential in older adults remains. As suggested by earlier research [37, 40], older adults might possess almost similar adaptability rates compared with younger ones, but the reduced recovery abilities place them at a higher risk of falling (i.e., relative deficit). Adaptation is a modification of a movement from trial to trial, which relies on error feedback [33, 90, 91]. There is consistent evidence that motor adaptation is mainly a function of the cerebellum and the cortico-cerebellar network [47–53]. The cerebellum uses efferent copies of the descending motor commands to predict future states [34, 47, 92]. Discrepancies between actual and predicted states generate an error signal that is then used to drive the adaptation process, by acting on the posterior parietal cortex and motor cortex to induce adapted movements [93]. However, with aging, changes in the cortico-cerebellar network were observed in imaging studies [56–58] and were suggested to reduce the locomotor adaptability over subsequent movements [94]. The basal ganglia and the cortico-striatal network also contribute to motor adaptation, particularly in the initial adaptation phase (exposure phase) [54, 55], most likely by selecting new sensorimotor representations that match the altered mechanical constraints more appropriately [94]. The frontal cortex areas then inhibit previous motor memories [95]. Likewise, an age-related degeneration of the frontal striatal-cortical network structures was reported [56, 59, 60, 96]. Neurophysiological structural impairments may explain well the smaller weighted averaged effect sizes of the general locomotor adaptability as well as predictive and reactive adaptation for the older adults compared with the young adults in the present meta-analysis. However, the meta-analysis and the included studies consistently showed that locomotor adaptability seems not to be significantly affected by aging. Therefore, it is possible that major adaptive functions remain preserved in older adults despite brain structural changes. This assumption is supported by findings of intact motor adaptation in older adults considering more simple movements [97–101] as well as postural tasks [102]. The neurophysiological basis for the preserved motor adaptation function of the older adults is yet unclear [94]; however, research suggests that brain/neural plasticity persists with aging, which may compensate for structural deficits [101, 103]. The present comprehensive analysis further showed that the adaptive potential of the older adults was present in a broad range of types of locomotion (i.e., trail and treadmill walking, sit-to-stand, gait initiation) and perturbations (i.e., slips, trips split-belt walking, obstacles, step target shifts), indicating that the neurophysiological basis of locomotor adaptability maybe more or less independent from the performed task.

The preserved locomotor adaptability is the basis for the design and application of effective intervention strategies targeting fall prevention. Training interventions that trigger the application of mechanisms responsible for an effective predictive and reactive dynamic stability control (e.g., modifications of the base of support or counter-rotating segments around the body’s center of mass [14]) using, e.g., repeated expected and unexpected movement perturbations as well as challenging environmental conditions, will progressively improve older adults’ stability performance [104–106]. Hence, the risk of falling can be notably reduced, indicating an effective and successful transfer from the interventions’ adaptation paradigm to the daily life condition [72, 74]. Furthermore, as unpredictable conditions may best approximate daily life situations, an implementation of new and unpredicted conditions is a promising approach to further consolidate the fundamental stability mechanisms and facilitate their variable application. Considering these different aspects in fall prevention interventions may efficiently reduce the age-related higher risk of falling and associated injuries.

The appropriate investigation of locomotor adaptability needs to take into account numerous methodological considerations (Table 1). The total methodological quality score in the present meta-analysis ranged from 56 to 90 %, with a mean of 67 ± 8 %, indicating adequate to high methodological quality for most studies and, thus, study validity with regard to the scope of the present research question (Electronic Supplementary Material, Table S1). However, several aspects of the internal study validity (i.e., study design, methods and cofactors) were not present in every study. First, only 12 of the 18 included studies reported values for the assessment of predictive adaptation and five for reactive adaptation and, therewith, allowed for a complete examination of the adaptive processes. To investigate predictive adaptability, it is necessary to quantify motor adjustments during a condition similar to the baseline and prior to/at the expected perturbation. However, this was not ensured in every study. For example, the split-belt paradigm considers the former criteria but violates the latter, because step or stride length asymmetry is detected during touchdown after the return to the baseline condition and, thus, already includes a certain reactive feedback response executed during swing phase. Furthermore, in several studies, an average value of the first steps was reported, increasing the abovementioned potential effect and, thus decreasing the discriminatory power of the respective study with regard to predictive and reactive adaptation. The appropriate assessment of reactive adaptation is even more challenging, as a wash-out phase (i.e., extinction training) to eliminate predictive adjustments needs to be included and the absence of predictive behavior must be controlled to ensure that the responses to subsequent unexpected perturbations are solely of reactive nature. Only one study accounted for these criteria adequately (Electronic Supplementary Material, Table S1). Moreover, only 12 of the 18 studies included a young-age control group. In consequence, the score for the internal validity was, on average, only 60 ± 16 %. Concerning the statistical validity, all studies applied appropriate statistical tests, but only one study calculated the effect size to estimate the effect of the perturbation-induced locomotor adaptions. The description of the experimental protocol and participants was appropriate in all studies, resulting in a high mean external validity score of 89 ± 6 %, although detailed information on the participants’ activity level, health status and cognitive status were mostly missing. The risk of bias assessment indicated low risk of all studies for the ‘outcome,’ ‘report’ and ‘other’ domains (Electronic Supplementary Material, Table S1). The ‘sequence’ and ‘allocation’ domain was not applicable since no group assignment was necessary for the present research question. However, if the assessor was blinded (‘blinding’ domain), e.g., in the analysis process, this was mostly not reported.

The present review and meta-analysis has some limitations. Only healthy older adults were included in the current article and, thus, a generalization of the present results to populations with different characteristics should be undertaken with care. For example, frailty [107] or diseases (e.g., Parkinson disease [108], cerebellar damage [34]) are likely to affect dynamic stability control and motor adaptability to different extents. Furthermore, the meta-analysis of reactive adaptation included only five studies, compromising the statistical power. Moreover, the present meta-analysis only considered studies in the English language.

## Conclusion

In conclusion, the present systematic review and meta-analysis provides evidence for a generally preserved locomotor adaptability and especially predictive and reactive adaptation in the elderly over a broad range of different locomotor tasks and mechanical perturbation kinds, with only minor, not statistically significant age-related deficits. These findings clearly emphasize the importance of training interventions targeting fall prevention. Respective interventions should implement the application of the mechanisms responsible for an effective predictive and reactive dynamic stability control in adaptation and learning paradigms to progressively improve older adults’ recovery performance.

Future studies may extend the present research question to specific populations (e.g., frail elderly, Parkinson disease patients) under consideration of the separate aspects of predictive and reactive adaptability. Furthermore, the effects of aging on the underlying neurophysiological mechanisms that are responsible for locomotor adaptation are not clear to date [i.e., age-related neurophysiological degeneration (structural decline) vs. persistent adaptability (functional preservation)] and need further clarification.

## Electronic supplementary material

Supplementary material 1 (DOCX 61 kb)
