# Supplementary material for: Predictive and Reactive Locomotor Adaptability in Healthy Elderly: A Systematic Review and Meta-Analysis
Source: Sports Med. 2015 Oct 20;45:1759–77. doi: 10.1007/s40279-015-0413-9 (PMC4656697; doi:10.1007/s40279-015-0413-9)
Supplement: Supplementary file 1 — Supplementary material 1 (DOCX 61 kb) [file 40279_2015_413_MOESM1_ESM.docx]

Electronic Supplementary Table S1. Methodological quality and risk of bias assessment of the included studies.

| Study | Methodological quality | | | | | | | | | | | | | | | | | | | | | | | | | | | | | | | | | | | | | e | Risk of bias | | | | | |
| --- | --- | --- | --- | --- | --- | --- | --- | --- | --- | --- | --- | --- | --- | --- | --- | --- | --- | --- | --- | --- | --- | --- | --- | --- | --- | --- | --- | --- | --- | --- | --- | --- | --- | --- | --- | --- | --- | --- | --- | --- | --- | --- | --- | --- |
|  | Internal  validity | | | | | | | | | | | | | | | | | Statistical  validity | | | External  validity | | | | | | | | | | | | | | | | Total  score  [%] |  | Sequence | Allocation | Blinding | Outcome | Report | Other |
|  | 1.1 | 1.2 | 1.3 | 2A | 2B | 2C | 2D | 2.1A | 2.1B | 2.2A | 2.2B | 2.2C | 3A | 3B | 3C | 3D | Score [%] | 4 | 5 | Score [%] | 6.1 | 6.2 | 7A | 7B | 7C | 7D | 7E |  | 8A | 8B | 8C | 8D | 8E | 8F | 8G | Score [%] |  |  |  |  |  |  |  |  |
| Bhatt et al., (2012) [72] | - | + | - | + | + | + | + | / | / | + | - | + | - | - | + | + | 53 | + | - | 50 | + | + | + | + | + | + | + |  | + | + | + | + | - | - | - | 89 | 64 |  | n.a. | n.a. | Unclear | Yes | Yes | Yes |
| Bierbaum et al., (2010) [16] | - | + | + | + | + | + | / | / | / | + | + | + | + | + | + | - | 79 | + | - | 50 | + | + | + | + | + | + | + |  | + | + | + | + | - | + | - | 93 | 74 |  | n.a. | n.a. | Unclear | Yes | Yes | Yes |
| Bierbaum et al., (2011) [17] | + | - | + | + | + | + | / | + | + | / | / | / | + | + | + | - | 79 | + | - | 50 | + | + | + | + | + | + | + |  | + | + | + | + | - | + | - | 93 | 74 |  | n.a. | n.a. | Unclear | Yes | Yes | Yes |
| Bohm et al., (2012) [36] | - | + | + | + | + | + | - | / | / | + | + | + | + | + | + | - | 75 | + | - | 50 | + | + | + | + | + | + | + |  | + | + | + | + | - | + | - | 93 | 73 |  | n.a. | n.a. | Unclear | Yes | Yes | Yes |
| Bruijn et al., (2012) [63] | - | + | + | + | + | + | / | / | / | + | + | - | - | - | + | + | 69 | + | - | 50 | + | + | + | + | + | - | + |  | + | + | + | + | - | + | + | 91 | 70 |  | n.a. | n.a. | Unclear | Yes | Yes | Yes |
| Chamber & Cham, (2007) [64] | - | + | + | / | + | + | - | / | / | + | - | - | - | - | + | - | 54 | + | - | 50 | + | - | + | + | + | + | + |  | + | - | + | + | - | + | - | 64 | 56 |  | n.a. | n.a. | Unclear | Yes | Yes | Yes |
| Hedel & Dietz, (2004) [112] | - | - | + | + | + | + | / | / | / | / | / | / | - | - | + | - | 45 | + | - | 50 | + | + | + | + | + | + | + |  | + | + | + | + | - | + | - | 93 | 63 |  | n.a. | n.a. | Unclear | Yes | Yes | Yes |
| Karamanidis et al., (2011) [81] | + | + | + | + | + | + | / | + | - | + | + | - | + | - | - | - | 77 | + | - | 50 | + | + | + | + | + | + | + |  | + | + | - | - | - | - | - | 82 | 70 |  | n.a. | n.a. | Unclear | Yes | Yes | Yes |
| Pai et al., (2014) [73] | - | + | - | + | + | + | + | / | / | + | - | + | - | - | + | + | 53 | + | - | 50 | + | + | + | + | + | + | + |  | + | + | + | + | - | - | - | 89 | 64 |  | n.a. | n.a. | Unclear | Yes | Yes | Yes |
| Pai et al., (2014) [74] | - | - | - | + | + | + | + | / | / | / | / | / | - | - | + | + | 30 | + | - | 50 | + | + | + | + | + | + | + |  | + | + | + | + | - | - | - | 89 | 56 |  | n.a. | n.a. | Unclear | Yes | Yes | Unclear* |
| Pai et al., (2010) [71] *a) walking* | - | - | + | + | + | + | + | / | / | / | / | / | - | - | + | + | 50 | + | - | 50 | + | + | + | + | + | + | + |  | + | + | + | + | - | - | - | 89 | 63 |  | n.a. | n.a. | Unclear | Yes | Yes | Yes |
| *b) Sit-to-stand* | - | - | + | + | + | + | + | / | / | / | / | / | - | - | + | + | 50 | + | - | 50 | + | + | + | + | + | + | + |  | + | + | + | + | - | - | - | 89 | 63 |  | n.a. | n.a. | Unclear | Yes | Yes | Yes |
| Pai et al., (2003) [37] | + | + | - | + | + | + | + | + | - | + | + | + | - | - | + | + | 71 | + | - | 50 | + | + | + | + | + | + | + |  | + | + | + | + | - | - | - | 89 | 70 |  | n.a. | n.a. | Unclear | Yes | Yes | Yes |
| Pavol et al., (2004) [40] | + | + | + | + | + | + | + | + | - | + | + | + | - | - | + | + | 86 | + | - | 50 | + | + | + | + | + | + | + |  | + | + | + | + | - | - | - | 89 | 75 |  | n.a. | n.a. | Unclear | Yes | Yes | Yes |
| Pavol et al., (2002) [46] | - | - | + | + | + | + | + | / | / | / | / | / | - | - | + | + | 50 | + | - | 50 | + | + | + | + | + | + | + |  | + | + | + | + | - | - | - | 89 | 63 |  | n.a. | n.a. | Unclear | Yes | Yes | Yes |
| Roemmich et al., (2014) [62] | + | + | + | + | + | + | / | + | - | + | + | - | - | - | + | - | 77 | + | + | 100 | + | + | + | + | + | + | + |  | + | + | + | + | - | + | - | 93 | 90 |  | n.a. | n.a. | Unclear | Yes | Yes | Yes |
| Sakai et al., (2008) [113] | - | - | - | + | + | + | / | / | / | / | / | / | - | - | + | - | 38 | + | - | 50 | + | + | + | + | + | - | + |  | + | + | + | + | - | + | - | 88 | 59 |  | n.a. | n.a. | Unclear | Yes | Yes | Yes |
| Tseng et al., (2010) [114] | - | - | + | + | + | + | / | / | / | / | / | / | - | - | + | - | 45 | + | - | 50 | + | + | + | + | + | + | + |  | + | + | + | + | - | + | - | 93 | 63 |  | n.a. | n.a. | Unclear | Yes | Yes | Yes |
| Yang & Pai, (2013) [61] | - | + | - | + | + | + | + | / | / | + | + | - | - | - | + | + | 53 | + | - | 50 | + | + | + | + | + | + | + |  | + | + | + | + | - | - | - | 89 | 64 |  | n.a. | n.a. | Unclear | Yes | Yes | Yes |
| **Mean±SD** |  |  |  |  |  |  |  |  |  |  |  |  |  |  |  |  | **60±16** |  |  | **53±11** |  |  |  |  |  |  |  |  |  |  |  |  |  |  |  | **89±6** | **67±8** |  |  |  |  |  |  |  |

**Methodological quality** (for detailed explanation of the criteria see table 1)**: 1 Study design** | 1.1 Reactive adaptability | 1.2 Predictive adaptability | 1.3 Young control group **| 2 Methods** | 2A Five trials for sufficient adaptive improvements | 2B Standardized perturbation | 2C Challenging perturbation | 2D Effect of security system **| 2.1 Reactive** | 2.1A Wash-out phase | 2.1B Effect of prediction **| 2.2 Predictive** | 2.2A Expectable perturbation | 2.2B Assessment of after-effects | 2.2C Purely predictive **| 3 Cofactors** | 3A Sex | 3B Activity level | 3C Health status | 3D Cognitive status **| 4 Statistical tests | 5 Power analysis | 6 Eligibility** | 6.1 Participants | 6.2 Variables **| 7 Description experimental protocol** | 7A Type of movement | 7B Movement characteristic | 7C Description perturbation| 7D Participant instruction | 7E Number of trials and blocks **|** **8 Description participants** | 8A Sex| 8B Age | 8C Body height | 8D Body weight | 8E Activity level | 8F Health status | 8G Cognitive status; The single criteria were rated ("+" = point, "-" = no point, "/" = not included) and used to calculate the quality score for each category (i.e. internal, statistical and external validity). The average of the three scores gives the total score. A white head of the table box indicates that a full point was assigned to each sub-category for the calculation of the score in the respective validity section ((assigned points / possible points)*100), whereas a grey head of the table box indicates that the sub-categories of the respective block were pooled to a single point (assigned points / possible points). **Risk of bias** [77]**:** **Sequence:** Adequate sequence generation, **Allocation:** Allocation concealment, **Blinding:** Blinding outcome assessor, **Outcome:** Incomplete outcome data, **Report:** Selective outcome reporting, **Other:** Other sources of bias; **Judgment:** Yes: low risk of bias, n.a. not applicable (only one cohort), Unclear: insufficient information reported (* baseline difference of control and training group regarding sex distribution and level of mobility).

Electronic Supplementary Material Appendix S1

Table: Search strategies

| **Database** | **Strategy** | **date** |
| --- | --- | --- |
| Web of Science (N=1127) | TS=("adaptation" OR "adaptive" OR "adaptational" OR "adaptability" OR "adjustments" OR "modifications" OR "responses") AND TS=("feedforward" OR "feedback" OR "proactive" OR "predictive" OR "reactive" OR "aftereffect" OR "after-effect" OR "after-effects") AND TS=(old* OR "aged" OR "age" OR "aging" OR "ageing" OR "senior" OR "elderly") AND TS=("walking" OR "walk" OR "gait" OR "run" OR "running" OR "sit-to-stand" OR "stand up" OR "transition" OR "stability" OR "split-belt") | 08.01.2015 |
| MEDLINE (N= 225) | ("adaptation, physiological"[MeSH Terms] OR "adaptation, biological"[MeSH Terms] OR „adaptive“[Title/Abstract] OR „adaptational“[Title/Abstract] OR „adaptability“[Title/Abstract] OR „adjustments“[Title/Abstract] OR „modifications“[Title/Abstract] OR „responses“[Title/Abstract]) AND („feedforward“[Title/Abstract] OR "feedback, physiological"[MeSH Terms] OR "feedback, sensory"[MeSH Terms] OR „proactive“[Title/Abstract] OR „predictive“[Title/Abstract] OR „reactive“[Title/Abstract] OR „aftereffect“[Title/Abstract] OR „after-effect“[Title/Abstract] OR „after-effects“[Title/Abstract]) AND (old*[Title/Abstract] OR "aged"[MeSH Terms] OR „age“[Title/Abstract] OR "aging"[MeSH Terms] OR „senior“[Title/Abstract] OR „elderly“[Title/Abstract]) AND ("walking"[MeSH Terms] OR „walk“[Title/Abstract] OR "gait"[MeSH Terms] OR „run“[Title/Abstract] OR "running"[MeSH Terms] OR "sit-to-stand"[Title/Abstract] OR "stand up"[Title/Abstract] OR „transition“[Title/Abstract] OR „stability“[Title/Abstract] OR „split-belt“[Title/Abstract]) | 08.01.2015 |
| EMBASE (N=386) | (ct=adaptation or ft=adaptive or ft=adaptational or ct=adaptability or ft=adjustments or ft=modifications or ft=responses) and (ft=feedforward or ct=feedback or ft=proactive or ft=predictive or ft=reactive or ft=aftereffect or ft=after-effect or ft=after-effects) and (ct=old or ct=age or ft=aged or ft=aging or ft=ageing or ft=senior or ft=elderly) and (ct=walking or ft=walk or ft=gait or ft=run or ft=running or ft=sit and stand or ft=stand and up or ft=transition or ft=stability or ft=split and belt) | 08.01.2015 |
| Science Direct (N=285) | (TITLE-ABSTR-KEY("adaptation" OR "adaptive" OR "adaptational" OR "adaptability" OR "adjustments" OR "modifications" OR "responses")) AND (TITLE-ABSTR-KEY("feedforward" OR "feedback" OR "proactive" OR "predictive" OR "reactive" OR "aftereffect" OR "after-effect" OR "after-effects")) AND (TITLE-ABSTR-KEY(old* OR "aged" OR "age" OR "aging" OR "ageing" OR "senior" OR "elderly")) AND (TITLE-ABSTR-KEY("walking" OR "walk" OR "gait" OR "run" OR "running" OR "sit-to-stand" OR "stand up" OR "transition" OR "stability" OR "split-belt")) | 08.01.2015 |

TS=Topic (Title+Abstract+Keywords), FT=free text, CT=controlled term
